# Is there a difference in joint line restoration in revision Total knee arthroplasty according to prosthesis type?

**DOI:** 10.1186/s12891-018-2295-0

**Published:** 2018-10-20

**Authors:** JuHong Lee, SungIl Wang, KiBum Kim

**Affiliations:** Department Of Orthopaedic Surgery, Chonbuk National University Hospital, 20, Geonji-ro, Deokjin-gu, Jeonju, 54907 South Korea

**Keywords:** Revision total knee arthroplasty, Joint line restoration, Offset stem, Posterior condylar offset

## Abstract

**Background:**

The aim of this study is (1) to compare joint line (JL) restoration and clinical outcomes in revision TKA based on the contemporary prosthesis type and (2) to determine the restoration of posterior condylar offset (PCO) according to the use of a femoral offset stem.

**Methods:**

Sixty knees that underwent revision TKA from April 2003 to December 2013 with a minimum of 1 year follow up were included. These were further subdivided into three groups according to prosthesis type: group I (2 mm offset), group II (4.5 mm offset), group III (2, 4, and 6 mm offset). The JL position change was defined as a change in the adductor tubercle distance, preoperatively versus postoperatively. We also collected the change of PCO in distal femur and clinical outcomes including range of motion (ROM) and knee scores at the preoperative and last follow-up periods.

**Results:**

The JL elevation for group III was significantly lower than that of the other groups. Usage of the tibial and femoral offset stem in group III was more frequent than in the other groups. PCO in revision TKA with a femoral offset stem was significantly greater than in those with a femoral straight stem. The JL position in revision TKA with a femoral offset stem was less elevated than in those with a femoral straight stem.

**Conclusions:**

More recent developed revision prosthesis with various sizes option of offset stem may be effective in restoring the native joint line as using the femoral offset stem more convenience in revision TKAs.

## Background

Increasing numbers of total knee arthroplasties (TKAs) are being performed, with excellent clinical results [1]. Survivorship of primary TKA has been reported to be 90% or greater at 10 to 15 years of follow-up [2–5]. However, a small percentage of patients suffer from inevitable conditions such as infection, aseptic loosening, polyethylene wear, and instability requiring revision TKAs. Overall, orthopedic surgeons will be encountering an increase in the incidence of revision TKA [1].

Joint line (JL) restoration is essential for primary as well as revision TKAs. Unfortunately, there is a natural tendency of JL elevation during revision TKAs [6, 7]. Excessive JL elevation may result in decreased range of motion (ROM) due to patella baja with impingement of the patella on the tibial component, anterior knee pain by increasing patellofemoral contact forces, increased component wear or extensor mechanism failure and mid flexion instability [7–9]. There are several recommendations to avoid JL elevation during revision procedures, including: (1) the size of the femoral component should be selected by medio-lateral remaining bone stock, not antero-posterior; (2) the flexion space should be kept as small as possible, as this results in a relatively larger flexion space after component removal and joint debridement, compared to the extension space; (3) a bone graft or metal augment should be used to reconstruct bone loss; (4) a constrained insert should be used in cases of extension-flexion gap mismatch [10]. Offset adapters, stem extensions and metal augments are commonly used to reconstruct the JL during revision TKA. The restoration of posterior condylar offset (PCO) is also important in conjunction with restoring the JL to achieve a balance between the flexion and extension gaps [11]. Furthermore, the restoration of PCO improves clinical outcomes including ROM [12].

Although the above-mentioned surgical techniques are theoretically employed, JL restoration in revision TKAs is difficult to achieve due to the variability of metal augments and offset systems according to the implant manufacturer. To date, there is a paucity of studies comparing JL elevation according to the type of contemporary prosthesis depending on the variability of metal augments and offset system.

Therefore, we hypothesize that there is difference in JL elevation according to the type of contemporary prosthesis and this difference will affect the clinical outcomes. The aim of the present study is (1) to compare JL elevation based on the type of contemporary prosthesis in revision TKAs, (2) to determine the restoration of posterior condylar offset (PCO) according to the use of femoral offset stem, and (3) to determine the association between clinical outcomes and the amount of JL elevation.

## Methods

### Patients

We retrospectively reviewed case series of 75 knees that underwent revision TKA from April 2003 to December 2013 with approval from the Institutional Review Board of Chonbuk National University Hospital. All consecutive revision TKAs were performed by a senior surgeon (JHL). We excluded seven knees that had incomplete clinical and radiologic data due to loss of follow up, six knees using valgus-varus constraint polyethylene (PE) insert, 1 re-revision knee and 1 knee using the structural allobone graft in order to control for JL position bias. Finally, 60 knees with a minimum of 1-year of follow up were enrolled in the present study. The mean duration of follow-up was 62 months (ranging from 12 to 96 months). Of the 60 knees, 38 were of female patients and 22 were of male patients. Etiologies in this case series were periprosthetic joint infections (PJIs) (48 knees), aseptic loosening (9 knees), instability (2 knees), and PE wear (1 knee).

The revision TKAs were sequentially performed using three different revision prostheses. The PFC® Sigma knee system (Depuy, Warsaw, Indiana) was used in 17 knees from July 2004 to February 2006, the NexGen® LCCK system (Zimmer, Warsaw, Indiana) was used in 13 knees from May 2006 to November 2010, and the Legion® total knee system (Smith & Nephew, London, UK) was used in 30 knees from May 2011 to December 2013. The three revision systems used in this study differed in the size of metal augment, femoral offset and modularity (Table 1). Demographic datas including follow-up period of the three groups are presented in Table 2.

**Table 1:** Characteristics of the revision prosthesis systemsd

	Group 1^a^	Group 2^b^	Group 3^c^
Femoral Offset	2 mm (Anterior or Posterior)	4.5 mm (360°)	2,4,6 mm (360°)
Tibial Offset	4 mm (360°)	4.5 mm (360°)	2,4,6 mm (360°)
Offset Adapter	x	x	o
Modularity^d^ (piece)	3	2	3
Femoral Augments	4, 8, 12, 16 mm	5, 10, 15, 20 mm	5, 10, 15 mm
Tibial Augments	10, 15 mm	5, 10, 15, 20 mm	5, 10, 15 mm

^a^PFC® Sigma knee system (Depuy, Warsaw, Indiana)

^b^NexGen® LCCK system (Zimmer, Warsaw, Indiana)

^c^Legion® total knee system (Smith & Nephew, London, UK)

^d^The number of pieces in each femoral and tibial component after using the stem and offset adapter

**Table 2:** Preoperative characteristics of each group

	Group 1 (*n* = 17)	Group 2 (*n* = 13)	Group 3 (*n* = 30)	*p-value*
Age (years)	67.9 ± 4.5	66.4 ± 6.9	72.1 ± 7.6	0.07
Sex (M/F)	7/10	3/10	12/18	0.52
Cause of revision				0.6
PJI	14(82.4%)	9(69.2%)	24(80%)	
Aseptic loosening	2(11.8%)	3(23.1%)	5(16.7%)	
Instability	0	1(7.7%)	1(3.3%)	
PE wear	1(5.9%)	0	0	
Bone defect				
Femur				
I	0	2(15.4%)	1(3.3%)	0.14
IIA	2(11.8%)	1(7.7%)	0	
IIB	15(88.2%)	9(69.2%)	25(83.3%)	
III	0	1(7.7%)	4(13.3%)	
Tibia				
I	11(64.7%)	7(53.8%)	12(40%)	0.42
IIA	2(11.8%)	4(30.8%)	10(33.3%)	
IIB	4(23.5%)	2(15.4%)	8(26.7%)	
III	0	0	0	
Knee scores				
KSKS	46.5 ± 10.7	38.7 ± 8.6	43.2 ± 11.2	0.12
KSFS	39.4 ± 13.4	33.8 ± 10.8	41.7 ± 13.9	0.21
WOMAC	84.4 ± 14.4	88.8 ± 7.0	87.8 ± 8.2	0.87
Range of motion(°)	104.7 ± 15.5	105 ± 28.9	95.3 ± 27.7	0.54
Weight (kg)	61.1 ± 12.6	59.5 ± 9.3	59.8 ± 13.7	0.79
Height(m)	1.55 ± 0.09	1.52 ± 0.05	1.54 ± 0.12	0.6
BMI (kg/m^2^)	25.3 ± 4.6	25.8 ± 3.0	25.2 ± 4.5	0.85
Follow up period (months)	66.1 ± 9.7	63.3 ± 10.3	60.7 ± 8.1	0.03

Data are presented as mean ± standard deviation or number (percentage), unless otherwise stated

### Operative technique

All revision TKAs for PJI were performed in two stages with the appropriate antimicrobial treatments, while surgeries for the other etiologies were performed in one stage. There was one re-revision case due to recurrence of PJI.

Similar to most revision TKAs, a standard medial parapatellar approach was performed in all procedures. When confronted with exposure difficulty due to tissue scarring or adhesion and patella baja, we conducted an additional procedure such as a rectus snip or tibial tubercle osteotomy. In this study, 5 cases required a rectus snip and 2 cases required a tibial tubercle osteotomy.

Femoral and tibial bone defects were assessed by the Anderson Orthopedic Research Institute (AORI) classification after removal of the original prosthesis [13]. Restoration of the native JL was the main goal with reconstruction of the tibia and femur by the prosthesis, including metal augments and offset systems. JL position was determined intraoperatively by measurement of the length from anatomical landmarks to the reconstructed JL. To obtain symmetry between the extension and flexion gaps, decreasing the flexion gap as much as possible was targeted by (1) using the larger femoral components unless an overhang of the mediolateral width of the remaining femoral bone stock was present, (2) a posterior translation of the femoral component whenever possible without notching the anterior femoral cortex.

Other intraoperative variables were also recorded such as grade of bone defects, thickness and constrained type of polyethylene, and whether bone grafts, metal augment and an offset stem of the tibia and femur were used or not. Finally, the prosthetic components were fixed with cement. The arthrotomy was closed in a routine manner. Identical post-operative management and rehabilitation protocol was conducted in all patients.

### Clinical and radiologic evaluation

**Fig. 1:**
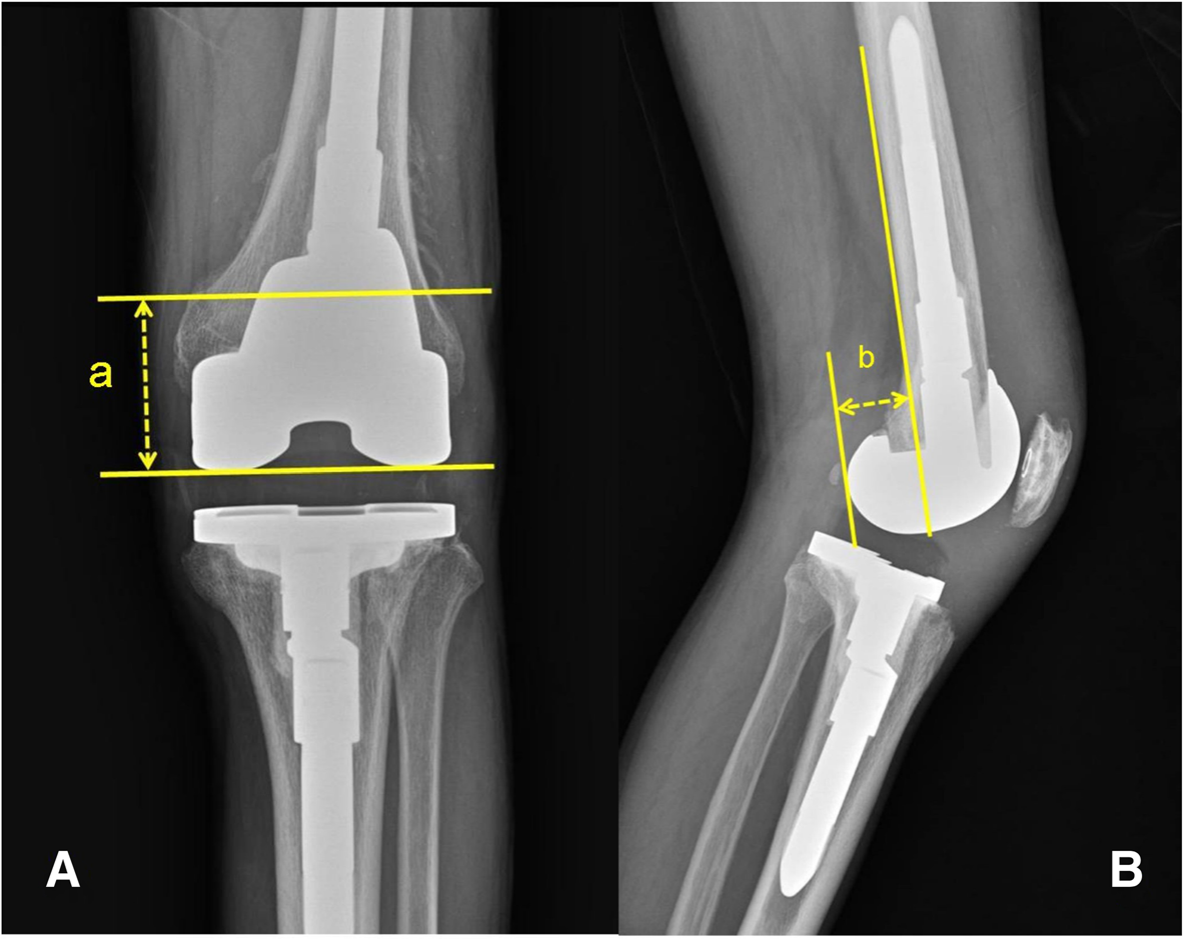
Radiologic measurement of the adductor tubercle distance (a) and the posterior condylar offset (PCO) of the femur (b) in a standing anteroposterior view (**a**) and a 30-degree flexion lateral view (**b**), respectively

For clinical evaluation, clinical data such as ROM, Knee Society knee score (KSKS), Knee Society function score (KSFS), and Western Ontario and McMaster Universities Osteoarthritis (WOMAC) index score were collected at the preoperative period and the last follow-up visit. Radiologic parameters including adductor tubercle distance (ATD), PCO and tibiofemoral angle were measured with the standing long leg anterior-posterior (AP) view and 30-degree flexion true lateral views of the knee with completely overlapping femoral condyles. ATD was defined as the perpendicular distance between the adductor tubercle and the most distal point on the medial supracondylar slope of the femur and the JL was defined as the tangent to the most distal points of the medial and lateral femoral condyles [14]. Figure1-A PCO was defined as the maximal thickness of the posterior condyle projected posteriorly to the tangent of the posterior cortex of the femoral shaft [15]. Figure 1-B To assess intra-observer reliability, the ATD and PCO were measured twice by one author (KBK), at a 1 month interval. To assess inter-observer reliability, a second author (SIW) measured the ATD and PCO.

In 56 cases with an unreplaced knee radiograph on the same or contralateral side, the change in JL position and PCO were defined as the difference between the ATD and PCO measured in the unreplaced knee radiograph and the postoperative ATD and PCO. Otherwise, in 4 cases without an unreplaced knee radiograph on the same or contralateral side, the change in JL was defined as the difference between the preoperative ATD and PCO on the contralateral replaced knee radiograph and postoperative ATD and PCO on the operated side.

Overall, knees were divided into three groups based on the three different prosthesis types, prosthesis had difference of direction and size option of offset stem (Group I - PFC® Sigma knee system, Group II - NexGen® LCCK system, Group III - Legion® total knee system).

The JL elevation, change of PCO, and clinical outcomes such as ROM and knee scores were compared among the three groups. We analyzed the correlation between change of JL, PCO and intraoperative variables to answer the question ``which is the effect about the restoration of JL and PCO during revision TKAs? ``.

### Statistical analysis

Repeatability and reproducibility of the radiologic measurements, such as ATD and PCO, were evaluated by calculating the intra-observer and inter-observer correlation coefficients. It was interpreted as poor if less than 0.4, marginal if greater than or equal to 0.4, but less than 0.75, and good when greater than 0.75. The difference in clinical outcomes after revision TKA was analyzed using a paired t-test. The radiologic and clinical variates among the three groups were analyzed by a Kruskal-Wallis test. The comparison of the change of JL and PCO according to the use of the femoral offset system was analyzed by a Mann-Whitney test. The correlation between change of JL, PCO and intraoperative variables was analyzed by Pearson’s correlation analysis. All statistical analyses were performed using SPSS for Windows, version 21.0 (SPSS, Chicago, Illinois). The level of significance was set at 5% (*p* < 0.05).

## Results

**Fig. 2:**
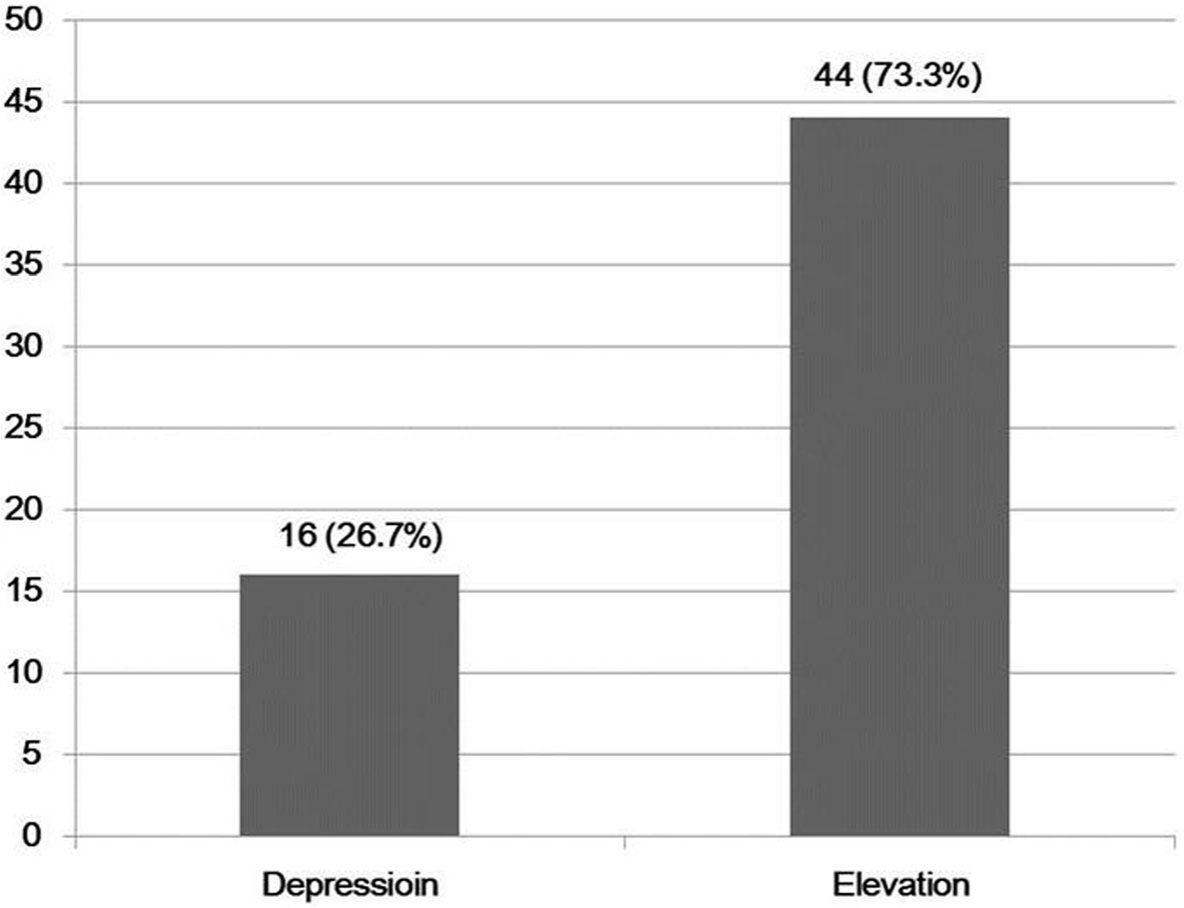
The distribution of JL position after revision TKA in all case series (A) and by group (B)

There was significant improvement in clinical outcomes, including ROM and knee scores (KSKS, KFKS and WOMAC), at the last follow-up visit Table 3. The JL position based on the change of ATD had a mean elevation of 2.3 ± 2.8 mm (ranging from − 3.92 mm to 9.92 mm). Fourteen knees (23.3%) were found to have a JL depression and 46 knees (76.7%) were found to have a JL elevation. Figure 2 Intraoperative variables among the three groups are delineated in Table 4. Both tibial and femoral stems were used more commonly in group III than in groups I and II. There was not a significant difference in the utilization of tibial and femoral augments, with the exception of distal lateral femoral augments.

**Table 3:** Clinical outcomes pre-operatively and at the last follow-up

	Pre-operative	Last follow-up	*P-value*
Tibiofemoral angle(°)	varus 0.2° ± 7.1°	valgus 4.6° ± 3.2°	0.00
Range of motion(°)	100.1 ± 25.2	108.8 ± 20.2	0.00
Flexion contracture(°)	6.6 ± 7.5	1.2 ± 2.5	0.00
Further flexion(°)	106.7 ± 21	109.9 ± 19.2	0.01
Knee scores			
KSKS	43.2 ± 11.2	86.7 ± 9.4	0.00
KSFS	39.3 ± 13.3	78.4 ± 9.7	0.00
WOMAC	87.1 ± 10.1	17.1 ± 7.5	0.00

Data are presented as mean ± standard deviation, unless otherwise stated

**Table 4:** Intraoperative variables, by group

	Group 1 (*n* = 17)	Group 2 (*n* = 13)	Group 3 (*n* = 30)	*P-value*
Femoral stem				
Offset/Straight (n)	9(53%) / 8(47%)	4(31%) / 9(69%)	29(97%) / 1(3%)	0.00
Size of offset (mm)	1.1 ± 1	1.4 ± 2.2	4.7 ± 1.7	0.00
Tibial stem				
Offset/Straight (n)	0(0%)/17(100%)	0(0%)/13(100%)	26(87%) / 4(13%)	0.00
Size of offset (mm)	0	0	3.7 ± 2.0	0.00
Metal Augments (n/size)				
Femoral Distal medial	16(94%) / 5.6 ± 2.5	9(69%) / 5 ± 4.1	26(87%) / 6 ± 3.3	0.16 / 0.38
Distal lateral	15(88%) / 5.4 ± 2.8	8(62%) / 4.6 ± 4.3	28(93%) / 6.5 ± 3.5	0.03 / 0.2
Posteromedial	17(100%) / 6.1 ± 2.1	11(85%) / 7.3 ± 3.9	24(80%) / 5 ± 3.2	0.15 / 0.08
Posterolateral	17(100%) / 6.1 ± 2.1	12(92%) /6.5 ± 3.2	24(80%) / 5.8 ± 4.0	0.11 / 0.49
Anterior	0 / 0	1(8%) / 0.4 ± 1.4	0 / 0	0.16 / 0.16
Tibial Medial	6(35%) / 4.1 ± 5.9	9(69%) / 5.8 ± 4.9	16(53%) / 4.5 ± 5.3	0.18 / 0.49
Lateral	4(24%) / 2.4 ± 4.4	2(15%) / 0.8 ± 1.9	8(27%) / 1.8 ± 3.6	0.72 / 0.67
Thickness of PE (mm)	15.9 ± 2.9	16 ± 2.6	14.9 ± 3.3	0.29

Data are presented as mean ± standard deviation or number (percentage), unless otherwise stated

JL position was elevated in all three groups. JL elevation in group III (1.2 ± 2.8 mm) was significantly lower than that of the other two groups (group I: 3.6 ± 2.6 mm, group II: 3.4 ± 2.3 mm). However, there was no significant difference in knee scores (KSKS, KFKS and WOMAC) and ROM among the three groups. The postoperative PCO was restored or increased relative to preoperative PCO in all three groups. The change in PCO was not significantly different between the three groups Table 5.

**Table 5:** Comparison of the radiologic measurements and clinical outcomes between the three groups

	Group 1 (*n* = 17)	Group 2 (*n* = 13)	Group 3 (*n* = 30)	*P-value*
Change of ATD (mm)	3.6 ± 2.6	3.4 ± 2.3	1.2 ± 2.8	0.00
Change of PCO (mm)	0.5 ± 2.6	0.8 ± 3.0	1.3 ± 2.2	0.58
Knee scores				
KSKS	87.7 ± 4.7	86 ± 16.4	86.5 ± 7.4	0.32
KSFS	77.6 ± 7.3	78.5 ± 13.8	78.8 ± 9.1	0.65
WOMAC	20.1 ± 9.4	15.6 ± 7.5	16.1 ± 6.0	0.26
Range of motion(°)	112.1 ± 12.4	110.4 ± 24.2	106.22.2	0.62
Flexion contracture(°)	0.3 ± 1.2	1.2 ± 3.0	1.7 ± 2.7	0.14
Further flexion(°)	112.4 ± 12.0	111.5 ± 21.9	107.8 ± 21.5	0.75

Data are presented as mean ± standard deviation, unless otherwise stated

Size of femoral offset stem among the intraoperative variables had a statistical significant correlation with change of JL and PCO, respectively (*r* = − 0.29, *r* = 0.32) Table 6. Based on these results, comparing the change of JL and PCO according to using femoral offset stem, the change of PCO in revision TKAs with a femoral offset stem was significantly greater than in those with a femoral straight stem. (*p* = 0.03) The JL position in revision TKAs with a femoral offset stem was statistically significantly less elevated than in those with a femoral straight stem. (*p* = 0.04) Table 7.

**Table 6:** Correlation coefficient (r) from Pearson’s correlation analysis between change of JL, PCO and intraoperative variables

Size	Change of JL		Change of PCO	
	r	*p*	r	*p*
Femoral offset	−0.29	0.03*	0.32	0.01*
Femoral anterior augment	0.33	0.12	0.12	0.37
Femoral distal medial augment	−0.12	0.37	−0.02	0.86
Femoral distal lateral augment	−0.17	0.2	0.02	0.91
Femoral posterior medail augment	0.2	0.12	0.3	0.02*
Femoral posterior lateral augment	0.17	0.21	0.3	0.02*
Tibial medial augment	− 0.07	0.59	−0.15	0.25
Tibial lateral augment	−0.05	0.71	−0.3	0.21

*: statistically significant

**Table 7:** Comparison of the change of ATD and PCO, by use of a femoral offset stem, regardless of prosthesis type

	Offset stem (*n* = 42)	Straight stem(*n* = 19)	*P-value*
Change of ATD (mm)	2.0 ± 3.0	3.4 ± 2.1	0.04
Change of PCO (mm)	1.5 ± 2.4	−0.4 ± 2.2	0.03

Data are presented as mean ± standard deviation

More than 5 mm change of JL was 9 knees (15%) in our case series. However, there was no significant difference in clinical outcomes between knees with 5-mm or greater JL elevation or depression and the others. Table 8.

**Table 8:** Comparison of the clinical outcomes between a 5-mm elevation or depression in JL position and the other else

	≥5-mm JL elevation or depression (*n* = 9)	The other else (*n* = 51)	*P-value*
Range of motion(°)	95.6 ± 25.8	111.1 ± 18.4	0.08
Flexion contracture(°)	1.7 ± 3.5	1.1 ± 2.3	0.77
Further flexion(°)	97.2 ± 22.7	112.2 ± 17.8	0.06
Knee scores			
KSKS	82.3 ± 19	87.5 ± 6.5	0.85
KSFS	73.9 ± 13.9	79.2 ± 8.7	0.35
WOMAC	21 ± 11	16.5 ± 6.7	0.24

Data are presented as mean ± standard deviation

The intra-observer and inter-observer correlation coefficients were 0.94 and 0.88 for ATD and 0.89 and 0.83 for PCO, respectively, confirming good repeatability and reproducibility of the ATD and PCO measurements.

## Discussion

The most important findings of the present study were that (1) the type of prosthesis used in group III was superior for restoring JL position although there was no significant difference in clinical outcomes between the three different types of prostheses, (2) using the femoral offset stem was beneficial for restoration of JL position by lessening the flexion gap, and (3) JL elevation or depression of 5 mm or greater did not deteriorate the clinical outcomes in this case series.

There are several methods available to determine JL position using plain radiographs. These methods typically use the distance between the JL and bony landmarks, such as the medial and lateral epicondyles, adductor tubercle, tibial tubercle and fibular head tip [16–18]. Various studies have reported the joint line position as an absolute value of these distances. However, recent studies have proposed that a ratio of these distances to the femoral width (the distance between the medial and lateral femoral epicondyles) has a theoretical advantage due to large variations between genders and different sized knees, making absolute values less useful [19, 20]. In revision TKA, bony landmarks are not always identified due to destruction by osteolysis and soft tissue scarring. In the present study, 57 of 60 knees were determined to have femoral bone defects greater than grade II and therefore the absolute value of the difference between the preoperative and postoperative ATD was inevitably used to determine the change of JL position.

The JL position in the present study was elevated by a mean of 2.3 mm and the PCO was enlarged by a mean of 1.0 mm after that revision TKA and clinical outcomes were comparable to previous studies of revision TKAs [21, 22]. These results were obtained using sufficient and appropriate metal augments (distal femoral augments in 55 (91.7%) of 60 knees, posterior femoral augments in 56 (93.3%) of 60 knees and femoral offset system in 42 (70%) of 60 knees). This resulted in restoration of PCO, and the JL position in group III was less elevated than in the two other groups.

We frequently encountered the difficult situations during revision TKAs and should solved these problems such as mismatch of metaphysis and diaphysis, unequal gap between extension and flexion space, asymmetry of mediolateral gap in flexion space to obtain stable and well functional reconstructed knee joint in revision TKAs [10, 23]. Offset stems were essential option to solve these problems in revision TKAs. Additionally, femoral offset stem had the theoretical advantages of restoration of PCO and JL [11]. In most revision TKAs, We encountered the larger flexion gap than extension gap. To avoid the elevation of JL, We should be lessened the flexion gap by using larger femoral component and downward femoral offset stem. Unfortunately, we could not use the offset stem even if we had to use it in complex revision TKAs due to notching of anterior femoral cortex. We thought that revision knee prosthesis commonly used was not patient specific prosthesis. Therefore, various sizes and universal direction (360 degree) of offset stem in revision knee prosthesis is important to be able to cope with complex and difficulty situations. Interestingly, we found that usage of a femoral offset stem was more frequent in group III than in the other two groups. The prosthesis used in group III was the most recent contemporary prosthesis with more options in the offset system in terms of size and modularity. This prosthesis might allow surgeons to use the offset system more readily in complex revision TKAs. Likewise, a tibial offset stem was also used more frequently in group III than in the other groups, which supports our hypothesis. More frequent usage of femoral offset stems may also be associated with the less elevated JL position in group III, compared to the other two groups.

JL restoration following primary and revision TKAs has been shown to affect clinical outcomes and patient satisfaction. In particular, JL restoration in revision TKAs yields significantly better results than when it is left unrestored by more than 5 mm [24]. A JL elevation greater than 8 mm results in better knee society scores than that of less than 8 mm [7, 25].

There was no association between the level of JL elevation and clinical outcomes in the present study, namely no difference in clinical outcomes was observed between the knees with JL changes of 5 mm or greater and the others. The predominant cause of revision TKA in the present study was PJI (80%). Septic revision TKAs have been shown to have inferior clinical results compared to aseptic revisions [26]. We thought that large proportion of septic revision TKAs in our cases may be affect the similar clinical outcomes regardless of JL elevation and prosthesis type.

There were several limitations in the present study. First, a relatively small sample size in each group was retrospectively reviewed. Second, unfortunately the native JL level could not be identified in 4 (6.7%) cases due to the absence of an unreplaced knee radiograph. We thought that it was serious limitation of this study, however, we enrolled and analyzed the 4 cases because it occupied the relatively small proportion of our case series. Third, the cause of revision was heterogeneous with PJI being the most common. Fourth, we classified femoral and tibial bone defects according to the AORI grading system, although this classification has limitation regarding the severity of bony defects. In particular, in grade II, the AORI grading system does not distinguish between mild to severe defects. This may affect the results showing no association between the femoral bone defects classified by the AORI grading system and JL elevation. Finally, we inevitably compared the clinical outcomes among the three group at last follow up period that was inhomogeneous period. It seemed to have the bias to interpret our results, however, mean follow-up period was comparable even though there were difference of follow up period among the three groups.

## Conclusion

More recent developed revision prosthesis with various sizes option of offset stem may be effective in restoring the native joint line as using the femoral offset stem more convenience in revision TKAs. Further studies with more patients and well-designed randomized controlled designs are required to confirm the results of the present study.

## Abbreviations

ATD: adductor tubercle distance
JL: joint line
KSFS: Knee Society function score
KSKS: Knee Society knee score
PCO: posterior condylar offset
ROM: range of motion
TKA: total knee arthroplasty
WOMAC: Western Ontario and McMaster Universities Osteoarthritis
